# Can Wearable Sensors Provide Accurate and Reliable 3D Tibiofemoral Angle Estimates during Dynamic Actions?

**DOI:** 10.3390/s23146627

**Published:** 2023-07-24

**Authors:** Mirel Ajdaroski, Amanda Esquivel

**Affiliations:** Department of Mechanical Engineering, College of Engineering and Computer Science, University of Michigan-Dearborn, Dearborn, MI 48128, USA; majdaros@umich.edu

**Keywords:** wearable devices, sports medicine, anterior cruciate ligament

## Abstract

The ability to accurately measure tibiofemoral angles during various dynamic activities is of clinical interest. The purpose of this study was to determine if inertial measurement units (IMUs) can provide accurate and reliable angle estimates during dynamic actions. A tuned quaternion conversion (TQC) method tuned to dynamics actions was used to calculate Euler angles based on IMU data, and these calculated angles were compared to a motion capture system (our “gold” standard) and a commercially available sensor fusion algorithm. Nine healthy athletes were instrumented with APDM Opal IMUs and asked to perform nine dynamic actions; five participants were used in training the parameters of the TQC method, with the remaining four being used to test validity. Accuracy was based on the root mean square error (RMSE) and reliability was based on the Bland–Altman limits of agreement (LoA). Improvement across all three orthogonal angles was observed as the TQC method was able to more accurately (lower RMSE) and more reliably (smaller LoA) estimate an angle than the commercially available algorithm. No significant difference was observed between the TQC method and the motion capture system in any of the three angles (*p* < 0.05). It may be feasible to use this method to track tibiofemoral angles with higher accuracy and reliability than the commercially available sensor fusion algorithm.

## 1. Introduction

There has been a discernible rise in non-contact anterior cruciate ligament (ACL) injuries among athletes, despite the implementation of preventive measures [1,2]. These injuries are of particular concern among young female athletes, with rates higher than their male counterparts in similar sports [1,2,3]. These injuries can be expensive to treat, with ACL reconstruction procedures ranging from USD 27,000 to USD 35,000 in cost; in addition, there is an increased risk of early-onset osteoarthritis of the knee following these operations [4,5,6]. Studies have identified sex-based differences in anatomy, hormones, and movement patterns as possible risk factors for ACL injury susceptibility [7,8,9,10,11,12,13,14,15,16]. While anatomical and hormonal factors cannot be controlled, identifying and modifying movement patterns that may contribute to ACL injury, or reducing exposure to severe loading cycles, is possible.

Traditionally, camera-based motion capture systems (MCS) are used in the laboratory to measure kinematics. These systems comprise multiple cameras and load cells which make them difficult to use ‘in the field’. An inertial measurement unit (IMU) is a wearable sensor made up of accelerometers, rate gyroscopes, and magnetic field sensors that measures the linear acceleration, angular velocity, and magnetic field strength of a particular body part. By using a specialized sensor fusion algorithm, the device can track the orientation of the body part in inertial space. Additionally, combining the IMU data of two adjacent body segments can be done to determine the Euler angles of a joint. These sensors have been utilized in different scenarios, including gait analysis and rehabilitation assistance, with success [17,18,19,20,21,22,23,24,25,26,27,28].

Research conducted in laboratory settings on cadaveric models has suggested that some ACL injuries are due to overuse and repetitive strain placed on the ACL [29]. Numerous scenarios can lead to these high-strain conditions; however, one theory that is commonly accepted suggests that such injuries occur when the knee is externally rotated with a slight flexion and a slight valgus angle at the time of the incident [13,30]. In theory, once these specific conditions are fulfilled, and the knee is subjected to a ground reaction force, a strong quadriceps contraction might occur, resulting in the tibia moving anteriorly to the femur; this movement could potentially cause the ACL to experience strain beyond its injury threshold [13,30]. The ground reaction and quadriceps forces, as well as the orientation of the knee, are crucial factors in this scenario [13,31]. Recently, a study has explored and created algorithms for calculating ground reaction forces with IMUs, with encouraging results. However, accurately estimating 3D tibiofemoral angles has been more challenging. In agreement with refs. [32,33,34,35,36], we believe that commercially available IMU algorithms may not be accurate for more dynamic activities like sports, where larger changes in joint angles, velocities, and accelerations can occur; this theory was corroborated through the results of our previous work [37]. Through testing with cadaveric specimens, we discovered that adjusting the settings of a sensor fusion algorithm led to improved performance compared to a commercially available version [37]. However, the use of cadaveric specimens excludes the effect soft tissue artifact may have on estimated angle accuracy. Thus, our goal in this study was to use the algorithm previously developed, but to adjust it for use in live human subjects and determine if the tuned algorithm demonstrates improved accuracy and reliability when measuring tibiofemoral (knee) angles in all three orthogonal directions during more dynamic activities. 

## 2. Materials and Methods

### 2.1. Subjects, Instrumentation, and Testing Procedures

**Figure 1:**
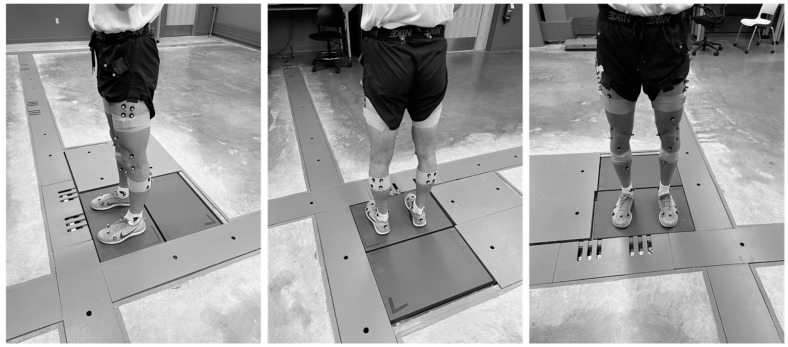
Marker and IMU locations.

Before the commencement of the study, we received IRB approval (HUM00150719). Nine healthy (5 females, 4 males) current or former (within two years) soccer or basketball athletes were recruited (Table 1). Participants with a BMI over 29 kg/m^2^ or who had a current lower extremity injury were excluded. Instrumentation and testing procedures have been described previously. Briefly, informed consent was obtained before a participant was fitted with 46 retro-reflective spherical markers; the first 30 markers were placed according to the 30 Rizzoli lower body protocol, with the remaining markers placed in clusters of four on the lateral thigh and gastrocnemius (Figure 1). Motions were collected using a 12-camera motion capture system (Prime 13 Optitrack, Corvallis, OR, USA) and two APDM wearable sensors (APDM Opal, APDM Wearable Technologies, Portland, OR, USA); the wearable sensors were placed similarly to previous works [37]. All data were collected at 200 Hz and a sync device was used to synchronize the recordings. IMUs were calibrated before each session through the predefined calibration conditions used by the Moveo Mobility software (Version 1.0) developed by APDM. Participants were asked to complete seven related tests three times: a jog with a pivot, an extension leap, a sidestep cut, a crossover cut, dominant shooting, a vertical jump onto two feet, and a vertical jump onto one foot (Table 2). Because both training and testing the validity of our tuned algorithm were needed, we randomly separated our subject pool based on average subject weight into a training set (used in tuning; N = 5) and a testing set (used in the testing of validity; N = 4).

### 2.2. Data Processing

For the MCS, a six-degree-of-freedom kinematic model of the lower extremity was created for each participant, including the pelvis, thigh, shank, and foot, using Visual 3D software (C-Motion, Germantown, MD, USA). A static trial was collected to determine the participant’s anatomic neutral. Joint angles at maximum ground reaction force were calculated in Visual 3D using a Cardan rotation sequence (CRS). Joint angles are the relative orientation of one local coordinate system to another and can be represented by three rotations about a unique axis. Emphasis is placed on the order of these rotations. Within this study, a CRS XYZ was implemented, with a lateral rotational matrix determined first, followed by an anterior, and finally a vertical rotational matrix; this process has been used in previous work [32]. Angles obtained through this kinematic model were normalized to the femur through the Visual 3D software. For the IMU, two methods were used to obtain the tibiofemoral angles: (1) the APDM proprietary algorithm (denoted as the IMU method); and (2) a tuned quaternion conversion algorithm (denoted as the TQC method). Angles obtained through the IMU method were not subjected to additional processing. 

IMU data were filtered using a fourth-order, zero-lag, low-pass Butterworth filter. Optimum cutoff frequencies for the Butterworth digital filter were obtained by applying Winter’s method; this method has been used in previous works [37,38]. Only the training set was utilized in determining the optimal frequencies (Table 3). Following filtering, a nine-axis indirect Kalman filter using the quaternion conversion of the IMU accelerometer, gyroscope, and magnetometer data was used. This process is detailed at greater length in our previous work and in the paper by Stanley et al. [37,39]. Briefly, this process began through the estimation of a sensor’s current orientations from the angular changes of the previous orientation with the initial alignment estimated to be north-east-down (NED). Once obtained, this estimated orientation was converted to a quaternion format. Next, quaternion conversion estimations of the gravitational and magnetic field measurements were obtained using linear acceleration and angular velocity. These estimates were used to correct the gravitational and magnetic field data obtained from the previous orientation and magnetometer. The corrected orientation and magnetometer estimates became the innovation for the indirect Kalman filter. As mentioned by Stanley et al., the indirect Kalman filter attempts to track errors rather than orientation as the data is updated through a recursive process [39]. Additionally, this process being recursive meant that the prior estimates of the error process and the state transition models were set to zero, thereby allowing for the application of the reduced Kalman equations [39]. Because orientation was in a quaternion format, the subtraction of the data from adjacent segments was mathematically valid. Therefore, the data attributed to the tibial IMU sensor was subtracted from the femoral IMU sensor. The resulting values were then converted into Euler angles, similar to what was done before [37].

### 2.3. Parameters Used in the TQC Method

Parameter adjustment followed what was previously done [37]. Briefly, several unique parameters used in the covariance of the observation model noise and the predicted estimate covariance were required; all the required parameters can be viewed at greater length in the work by Stanley et al. [39]. APDM Opal documentation provided the values for accelerometer noise, gyroscope noise, and magnetometer noise. Gyroscope drift noise, linear acceleration noise, and magnetic disturbance noise were not detailed in the documentation, so the default values based on the FRDM-FXS-Multi family of sensor boards (as used by the Freescale Toolbox of MATLAB) were used; this was done in our previous work [37]. The use of these default values was implemented due to the potential value range being theoretically infinite, such that a process of trial and error would not be possible.

The linear acceleration decay factor (LADF) and magnetic disturbance decay factor (MDDF) were unknown parameters that accounted for the effects of drift in either the linear acceleration or magnetic disturbance. Because their theoretical values ranged from 0 to 1, and because, in previous testing, differences less than 0.0001 resulted in no distinguishable change between subsequent LADF/MDDF, the values for the LADF/MDDF were theoretically finite. Using the training set of data, a trial-and-error approach was taken where adjustments to the LADF and/or MDDF were performed in increments of 0.0001 from 0.0001 to 0.9999 to minimize the difference in angles between the TQC method and the MCS. These adjustments were performed on each axe, resulting in 3 unique LADF and 3 unique MDDF values per trial. An average for both the LADF and MMDF was determined and used when calculating the angles of the testing set for validation. A trial-and-error approach has been used in previous work [37]. 

### 2.4. Statistical Analysis

A repeated measured ANOVA with a pairwise comparison was performed on both the training and testing sets to determine whether there were differences between the TQC method and the MCS and between the IMU method and the MCS. A Bonferroni correction was used to correct for multiple comparisons. Differences were considered significant if *p* < 0.05. Bland–Altman (BA) limits of agreement (LoA) were constructed for the testing set to assess each method’s reliability. LoAs were taken as the 95% confidence interval of the residual difference between a sensor method (either the TQC or the IMU) and the MCS. To assess each method’s accuracy, the root mean square error (RMSE) was determined between the MCS and either the IMU or TQC methods. BA plots were developed for descriptive analysis and used to comment on trends/biases that may exist; this was based on whether angles estimated using the IMU exhibited a tendency to over/underestimate true angles, or whether there was an increase or decrease in variability as the angle increased in magnitude.

## 3. Results

### 3.1. Training Set

**Figure 2:**
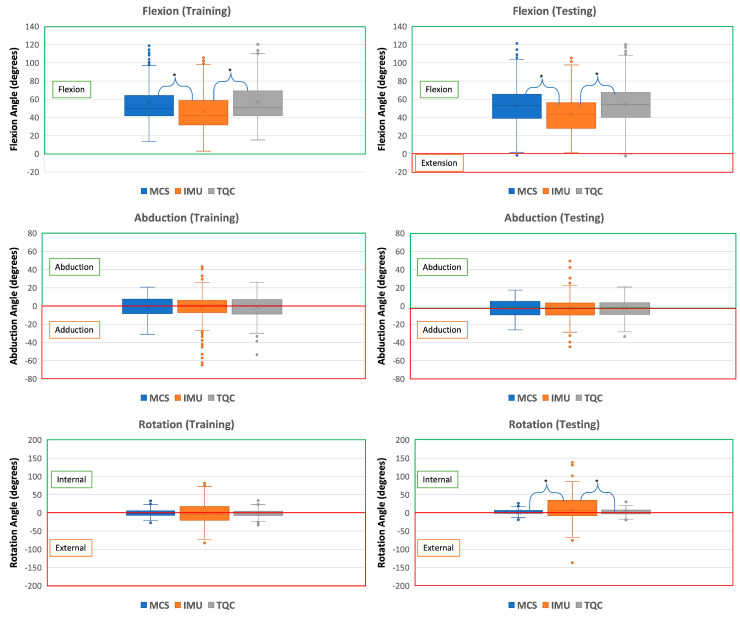
Box-and-whisker plots comparing the values of the motion capture system (MCS), the sensor fusion algorithm of the IMU (IMU), and the tune quaternion conversion method (TQC) for flexion (**top**), abduction (**middle**), and rotation (**bottom**). Differences that were determined to be significant are denoted by *.

Among all comparisons within the training set, flexion angle differences between the MCS and IMU, as well as between the IMU and TQC, were the only ones that were statistically significant (Figure 2). The training set showed a mean difference in flexion/extension between the MCS and IMU of −9.94° (95% CI: −13.9° to −5.96°), while that between the MCS and TQC was −0.18° (95% CI: −4.16° to −3.80°). For abduction/adduction, a mean difference of −0.47° (95% CI: −2.71° to 1.77°) between the MCS and IMU was observed, while the mean difference between the MCS and TQC was −0.13° (95% CI: −2.37° to 2.11°) (Figure 2). The mean difference in rotation between the MCS and IMU was −0.71° (95% CI: −4.01° to 2.60°); between the MCS and TQC, the difference was −0.10 (95% CI: −3.41° to 3.21°). Across all cases, the LoA and the RMSE of MCS vs. TQC were determined to be smaller, indicating increases in both accuracy and reliability in the TQC (Table 4).

### 3.2. Testing Set

Flexion and rotation angles between the MCS and IMU, as well as between the IMU and TQC, were determined to be statistically significant (Figure 2). A mean difference in flexion/extension of −9.96° (95% CI: −14.3 to −5.64) was determined between the MCS and IMU, while that between the MCS and TQC was determined to be 0.80° (95% CI: −3.52 to 5.12). For abduction/adduction, the mean difference between the MCS and IMU was −0.68° (95% CI: −2.58 to 1.23), while the mean difference between the MCS and TQC was −0.49° (95% CI: −2.39 to 1.42) (Figure 2). For rotation, the mean difference between the MCS and IMU was 5.42° (95% CI: 1.68 to 9.17), and it was 0.04° (95% CI: −3.70 to 3.79) between the MCS and TQC. Across all comparisons, the differences between the MCS and IMU resulted in larger RMSE values as well as larger LoAs (Table 4).

**Figure 3:**
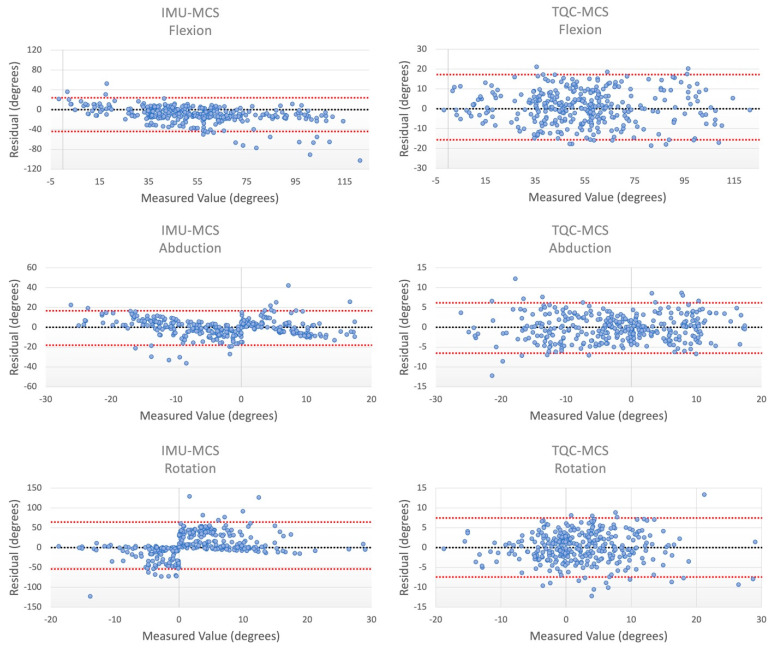
Bland–Altman plots associated with the residuals for flexion (**top**), abduction (**middle**), and rotation (**bottom**) between the motion capture system (MCS) and either the sensor fusion algorithm (IMU; **left**) or the tuned quaternion conversion method (TQC; **right**). Residuals were plotted against the measured values of the MCS. Limits of agreement (LoAs) are shown in red while the line of zero difference is shown in black.

The BA plots comparing the MCS and IMU differences showed varying degrees of bias and trends. For flexion/extension, there tended to be an underestimation of the angle by the IMU, and a linear slope was observed between the residuals and the measured flexion/extension angle; lower measured values skewed towards overestimations while higher values tended to be underestimations (Figure 3). Within the abduction/adduction BA plot between the MCS and IMU, as the magnitude of the measured angle increased (regardless of whether abduction or adduction), estimated values by the IMU decreased (Figure 3). For rotational angles between the MCS and IMU, the IMU tended to overestimate the magnitude of the angle, regardless of whether it was internal or external rotation. Comparing the MCS and the TQC, differences in flexion/extension as well as rotation showed no discernable trends. With the abduction/addiction BA plot between the MCS and IMU, as the magnitude of the measured angle increased, there was an increase in the variability of TQC estimates.

## 4. Discussion

We hypothesized that the sensor fusion algorithm employed by commercially available wearable sensors may not necessarily be accurate when used in dynamic activities, such as sports where larger changes in joint angles, velocities, and accelerations occur. Previous cadaveric testing supported this theory, as the tuning of certain parameters of a quaternion conversion algorithm resulted in more accurate and reliable angle estimates over an IMU’s sensor fusion algorithm [37]. However, we recognized several limitations in that previous work, such as a lack of soft tissue and a lack of variability in the motion. The purpose of this study was to use the algorithm previously developed but to retune it for use in human subjects in a variety of action types and determine if this tuned algorithm demonstrated improved accuracy and reliability in angle estimations. In all cases for the testing set, the differences between the TQC and MCS were determined to be statistically insignificant, while differences between the IMU and MCS were observed as being significant in both flexion and rotation. Whether or not these differences are clinically relevant is dependent on the particular scenario. A review paper examining ACL tears in athletes reported flexion angle differences of 10°–21.7° between injured and uninjured knees, and a laboratory study observed abduction angle increases of 8° in athletes who went on to injure their ACL [8,40]. Therefore, while differences between the IMU and MCS were observed as being statistically significant in flexion, clinically this may be negligible. Across all comparisons, the RMSE values associated with the differences between the TQC and MCS were smaller than those between the IMU and MCS, with the largest difference occurring in the testing set rotational angles (3.63° compared to 29.7°); this was similarly observed during our cadaveric testing [37]. In every case, the LoA associated with the difference between the TQC and MCS were smaller than those between the IMU and MCS, indicating a smaller range in differences and a better degree of reliability. 

Various studies have calculated joint angles, particularly those of the knee, using IMU-derived data, and then compared the results to those obtained through an MCS [19,33,34,35,36,41,42,43]. However, the focus of many of these studies has been on examining differences in flexion angles, possibly due to the knee flexion angle exhibiting the largest change during an action, and consequently being easiest to measure reliably. However, being able to accurately measure abduction and rotation angles is important because of the potential effects these angles may play in knee injuries, specifically, in ACL injuries. Many studies have proposed there is a correlation between ACL strain and the abduction/adduction and rotational orientation of the joint [16,44,45,46,47,48,49,50]. Because of this possible correlation, to improve knee injury tracking, it becomes important to track all three knee angles. Furthermore, the method by which joint angles are determined through IMUs also varies widely from study to study, with some taking the integration of angular rates while others use Euler angles. Studies such as those by Watanabe et al., Tong et al., and Bakhshi et al. calculated knee joint angles by taking the integration of angular rates, without the conversion to Euler angles and limiting themselves to only determining flexion angles [19,34,35]. This process may be problematic as angles obtained do not describe the orientation of the joint to a fixed coordinate system, but rather to some arbitrary coordinate system. As such, each study’s estimated angles may not represent the knee’s true orientation, and comparisons between estimated and MSC-measured values may not be accurate. However, these studies have shown their methods to be accurate in knee flexion estimates as the least accurate of these studies (Tong et al.) demonstrated an RMSE value below that which we reported between the MCS and TQC (our best case within flexion comparisons) (RMSE of 6.4° compared to 7.48°) [35]. This comparison may warrant criticism, as these studies observed actions associated with rehabilitation, which can be less dynamic than those in our study, but it may be that, due to possible increases in both angular velocities and linear accelerations, the results of our study were more prone to noise pollution and thus inaccuracies. Various studies have noted the potential effects of soft tissue artifact on the validity of results [51,52,53]. 

Bell et al. and Zügner et al. compared angles estimated via a sensor’s proprietary sensor fusion algorithm and those of an MCS [41,42]. In these cases, the sensor fusion algorithm was used to calculate the Euler angles of the knee joint. In the study by Bell et al., RMSE values between estimated and measured knee flexion angles were lower than any previously mentioned study and lower than that of our study (RMSE of between 2° and 2.9° compared to 7.48°) [42]. While this shows a high accuracy for flexion angle estimates, the accuracy of abduction/adduction or rotational estimates were not reported, which are important if IMUs are to be used for injury prevention. Zügner et al. reported that they found no significance in the mean difference between estimated and measured knee flexion angles (*p* = 0.7) and determined a high ICC (ICC > 0.8) [41]. Mean difference comparisons and ICC compare data sets as groups and, as such, mitigate the effects of gross differences; if the algorithm over- and underestimates angles similarly, the mean difference would be relatively small and may be misleading to the performance of an algorithm. Approaches such as RMSE or mean absolute percent errors remove this pitfall and may be much better indicators of accuracy. 

In a previous study performed using APDM Opal sensors, highly dynamic activities (jumps) were observed and comparisons of all three knee angles were reported [37]. It was concluded that, for both abduction/adduction and rotation, the APDM Opal algorithm was able to perform well under certain conditions, particularly those of smaller angle displacements, and experienced greater variability as the magnitude of the measured angle increased [37]. We observed a very similar trend when comparing the IMU to the MCS across both abduction/adduction and rotation angles, but it was only within the abduction/adduction comparison between TQC and MCS that a similar trend was noted; however, the extent of the increase in the variability as the angle magnitude increased was not as prominent. In both this and our previous study, the mean difference between the IMU estimated abduction/adduction angle and that of the MCS was not significant, although the RMSE reported in this study was greater (RMSE of 8.59° compared to 4.91°) [37]. However, several factors may have influenced this, such as the limited range of action previously observed (limited to only vertical jumping). Differing action types, including cutting maneuvers that induce more abduction/adduction and rotation, were examined in this study. Normalization of both RMSE values may provide a better means of comparison. Across all cases, the RMSE values between the TQC and MCS were smaller than those reported by our previous study, illustrating that perhaps the TQC may estimate angles more accurately. Furthermore, comparing the BA plots showed that the TQC demonstrated an overall greater degree of reliability, as both lesser trends and smaller LoAs were observed. 

### Limitations

This study had several limitations, including the use of a controlled laboratory setting. Due to the limited space and potential lack of comfort, participants may not have been able to perform actions exactly as they would on-field. The floor, being stiffer than the common surfaces in sports, might have also impacted our results, as several studies have tracked the effects of surface stiffness on energy absorption and biomechanics; it may be possible that greater errors/variations would be present in on-field activities where surface stiffness can vary greatly [54,55]. The small population size (N = 9) of our study was a limitation. Additional participants with more varying age, weight, height, etc., may be needed to assess the true accuracy and reliability of the TQC. The way our sensors were affixed may also be a limitation for using this in the field, as on-field use of these sensors may preclude them from being similarly placed on athletes. Either due to game rules or athlete comfort, sensor placement may need to be adjusted. Studies conducted have correlated the relationship between sensor position and orientation with errors in estimations, showing a reduction in accuracy of up to 20.8% [56]. Thus, although when using the current sensor placement we reported high levels of accuracy and reliability when implementing the TQC, changes in sensor orientation/placement may adversely affect the results. Additionally, the duration of each test may have omitted, or greatly reduced, the potential effect drift and error accumulation can have on angle estimates. While the TQC accounts for gyroscopic drift in a recursive process, test actions were performed within several seconds. During on-field implementation, the sensor may need to be recording for upwards of two hours, depending on the sport being examined. The effects of prolonged recording on the accuracy/reliability of the TQC warrants additional research.

## 5. Conclusions

Our goal in this study was to ascertain the potential improvement in accuracy and reliability of a tuned quaternion conversion algorithm when compared to the sensor fusion algorithm often provided by manufacturers. The theory was that, due to their popularity in rehabilitation settings, various parameters within the sensor fusion algorithm are tuned to less dynamic actions and therefore may not be robust enough for implementation in sports-related activities. Tuning a quaternion conversion algorithm and using dynamic actions, we recorded improvement across all three angle types, as the TQC was able to more accurately (lower RMSE) and more reliably (smaller LoA) estimate an angle than the IMU. It may be feasible to use this algorithm to identify and track knee angle measurements that might be associated with ACL injury. However, further testing for the effects that surface-of-play can have, the optimal placement of sensors to ensure adherence to rules and player comfort, and the effects that drift may have on the prolonged recording, will be needed before an on-field application is possible.

## Figures and Tables

**Table 1:** Demographics of the subjects, separated between those used in tuning the algorithm and those used in validation. Sport denotes the sports a subject participated in, with dual denoting a subject who had played both sports within the allotted time.

**Demographics of the Subjects Used in Amendment Training**				
**Subject ID**	**Sex**	**Mass (kg)**	**Height (m)**	**Sport**
TR1	F	79.0	1.85	Soccer
TR2	F	69.0	1.78	Basketball
TR3	F	63.2	1.63	Soccer
TR4	M	78.5	1.83	Soccer
TR5	M	70.3	1.78	Soccer
**Demographics of the Subjects Used in Amendment Testing**				
**Specimen ID**	**Sex**	**Mass (kg)**	**Height (m)**	**Sport**
TE1	F	75.4	1.75	Dual
TE2	M	74.9	1.85	Soccer
TE3	F	63.7	1.73	Basketball
TE4	M	85.8	1.83	Soccer

**Table 2:** Actions performed by subjects along with descriptions. Note: dominant shooting was only performed by individuals who have played soccer.

Movement	Description
Jog with pivot	Participant jogs a few steps and pushes off dominant leg to turn body towards the opposite side.
Extension leap	Participant pushes off dominant leg while fully extending the non-dominant leg to land on force plate more than two comfortable step lengths away.
Sidestep cut	Subject accelerates toward the direction opposite of the planted leg.
Crossover cut	Participant crosses one leg over the planted leg and accelerates in the direction of the push off leg.
Dominant shooting	Non-dominant foot takes a firm step onto force plate to support the dominant leg swing through a “shooting” motion (Soccer or Dual subjects only).
Jump—land on one foot	Subject stands with feet together, jumps maximum height, and lands on one foot.
Jump—land on two feet	Subject stands with feet together, jumps, and lands on two feet at maximum height, 50% of maximum and 25% of maximum.

**Table 3:** The optimal cutoff frequencies determined through the application of Winter’s method for each direction. The average of both the right and left legs is presented. All filtering frequencies are in Hz and based on a sampling rate of 200 Hz.

Optimal Cutoff Frequencies Determined for the IMUs				
Parameter		X	Y	Z
Tibial IMU	Linear Acceleration	19	20	18
	Angular Velocity	18	18	22
	Magnetometer	7	7	7
Femoral IMU	Linear Acceleration	17	14	16
	Angular Velocity	17	19	15
	Magnetometer	7	7	7

**Table 4:** Comparisons of the angles estimated using either the IMU or TQC method to those of the MCS. Presented are the RMSE and LoA bound (taken as the difference between upper and lower bound). Diff. is taken as the difference of MCS vs. TQC values or MCS vs. IMU.

Comparisons of the IMU and TQC Methods to MCS					
		Training Set		Testing Set	
	Comparison	RMSE	LoA (Upper–Lower)	RMSE	LoA (Upper–Lower)
Flexion	MCS vs. IMU	16.6°	52.0°	19.0°	68.1°
	MCS vs. TQC	7.48°	29.3°	8.00°	32.9°
	**Diff.**	**−9.08°**	**−22.6°**	**−11.0°**	**−35.2°**
Abduction	MCS vs. IMU	11.9°	46.6°	8.59°	33.4°
	MCS vs. TQC	3.51°	13.8°	3.14°	12.9°
	**Diff.**	**−8.37°**	**−32.8°**	**−5.44°**	**−22.4°**
**Rotation**	MCS vs. IMU	25.5°	100°	29.7°	121°
	MCS vs. TQC	4.39°	17.2°	3.63°	15.0°
	**Diff.**	**−21.2°**	**82.9°**	**−26.1°**	**105°**

## Data Availability

The data presented in this study are openly available in Esquivel, A., Ajdaroski, M. Using Wearable Sensors to Provide 3D Tibiofemoral Angle Estimates during Dynamic Actions Data Set [Data set], University of Michigan—Deep Blue Data.
